# Demographic and circumstantial accounts of burn mortality in Cape Town, South Africa, 2001-2004: An observational register based study

**DOI:** 10.1186/1471-2458-9-374

**Published:** 2009-10-06

**Authors:** A Van Niekerk, R Laubscher, L Laflamme

**Affiliations:** 1 Crime, Violence and Injury Lead Programme, Medical Research Council-University of South Africa, Cape Town, South Africa; 2 Biostatistics Unit, Medical Research Council, Cape Town, South Africa; 3 Karolinska Institutet, Department of Public Health Sciences, Division of International Health, Stockholm, Sweden

## Abstract

**Background:**

Burns are a persisting public health problem in low- and middle-income countries; however, epidemiologic data for these settings is scarce. South Africa is no exception although there is an emerging knowledge base, especially for paediatric burns. The current study describes the epidemiology of burn mortality across the lifespan in Cape Town (2.9 million inhabitants in 2001), one of the six South African metropolitan centres.

**Methods:**

The distribution of burn mortality across socio-demographic groups and also their circumstances of occurrence were investigated using four year (2001 to 2004) surveillance data from the National Injury Mortality Surveillance System (n = 1024 cases).

**Results:**

Burn mortality occurred at a rate of 7.9 per 100 000 person-years (95% CI: 7.3-8.3). Males sustained fatal rates 2.2 times more than that for females (p < 0.001), with rates significantly higher in the 25 to 38 and 39 to 50 age groups than at other ages (p < 0.001). The greatest difference between male and female deaths was observed in the 25 to 38 year age group, when almost three male deaths occurred for every female one. The vast majority of fatal burns were registered as accidental and occurred in the home, either over the cold and wet months or during recreational periods over weekends and across the year. Alcohol intoxication was reported for the majority of those adults whose alcohol blood levels were tested (i.e. 52.6% of cases aged 16+ years).

**Conclusion:**

Besides paediatric burns, the high prevalence and circumstances of occurrence of burns among middle age men are a source of concern. There are reasons to believe that this over-representation is a reflection of detrimental living conditions, life-style and poor socio-economic status. It is recommended that there be greater prioritisation of prevention activities that involve the control or management of kerosene heat sources, the provision of alternatives to flammable housing materials, and the implementation of strategies to reduce harmful drinking practices.

## Background

The World Health Organization (WHO) estimates that each year over 300 000 people die from flame or fire-related burn injuries, but this excludes deaths as a result of scalds, electricity, chemical burns and other forms of burn injury, about which less is known [1]. There is no indication of the extent of global burn morbidity or hospitalizations across the lifespan, although over half a million paediatric hospitalizations are estimated to occur each year [2]. Burns, as is the case for most causes of injuries, are disproportionately concentrated in low- and middle-income countries (LMICs) [3]. In 2001, the rates of fire mortality in LMICs were 4 per 100 000 persons and 7 per 100 000 persons for males and females, respectively. Overall, these rates were around 9 times higher than for high-income countries (HICs) (4.53 compared to 0.51 per 100 000) [3]. Fire-related mortality rates are especially high in South East Asia (11.6 deaths per 100 000); but also high in the Eastern Mediterranean (6.4 per 00 000) and Africa (6.1 per 100 000) [1,3].

In HICs much has been done to lower the extent and impact of fires and burns, largely through sustained research on the descriptive epidemiology and risk factors to injury and mortality, the implementation of legislation and interventions to reduce risk exposure (including smoke detector promotion programmes, tap water temperature reduction, and parent and child education), and parallel burn care interventions [1,4,5]. In LMICs, research that accurately describes the magnitude, risks and costs of burns is available for a number of countries, but often more limited in scope, due to such factors as data limitations. LMIC descriptions of burns have highlighted the vulnerability of children, and identified selected individual, technological and social risk factors [1,4,6].

South Africa is one of a number of LMICs for which there is an emerging platform for burn prevention. South African research has recently described the extent of urban burn mortality [7]; child burn morbidity and patterns and circumstances of occurrence [6,8]; the contribution of neighbourhood poverty, housing conditions and child dependency on child morbidity [9]; and perspectives on the aetiology and prevention of childhood burns [10]. These investigations have contributed towards initial indications of the extent of burn mortality and more synthesized descriptions of the demographics and circumstances of child burn morbidity in resource poor settings [11]. There, however, remains limited fatal or non-fatal burn descriptions across the lifespan, with comprehensive burn mortality data only available for the four largest South African metropolitan centers; of these, the city of Cape Town, has reported the highest burn mortality rates [7].

LMIC settings remain affected by persisting constraints to resource mobilization, interpersonal, cultural and technological capacities, which inhibit the development or implementation of interventions to control exposure and trauma outcomes [1,4]. In addition, adult burn mortality has been a neglected public health issue, in South Africa and across the continent, due to the scarcity or inadequacy of empirical data and the greater emphasis on maternal and child health threats [12]. An epidemiological basis that depicts the extent and the nature of burn mortality is essential for the allocation of resources and the development of both prevention and trauma response interventions [1,4,5]. [4]. Comprehensive and detailed information on the demographics and circumstances of burn mortality in South Africa has yet to be described [4].

The aim of this study was to address this knowledge gap and provide an analysis of burn mortality across all ages in Cape Town. It investigates how burn mortality is distributed across age groups, sex and population groups and also what the typical circumstances of burn mortality occurrence are.

## Methods

The current study investigated burn mortality data recorded by the National Injury Mortality Surveillance System (NIMSS). The study is an observational register-based analysis of burn mortality for Cape Town over the four year period, 2001 to 2004.

### Data sources

#### Injury data

The NIMSS produces and disseminates descriptive epidemiological information on deaths due to non-natural causes, which are subject to a medico-legal autopsy and which by law must be followed for all deaths known or suspected to have arisen from unnatural causes, irrespective of whether these are referred via the public or private health sector [7,13,14]. Information for this system is collected by the police and forensic pathologists at each mortuary, and captured soon after the post-mortem, usually while the body is still located at the mortuary. This information is entered into a computerised database by mortuary or NIMSS administrative staff. The NIMSS records the deceased person's age, sex, population group, province, town and suburb of injury, scene of injury, and apparent manner and circumstances (or external cause) of death [13,14]. Temporal data is recorded, as is the presence of alcohol or any other substances in the deceased through information from forensic laboratory reports.

The NIMSS had, at the time of this study, full coverage for a number of South African cities, including Cape Town [7]. The NIMSS collates burn deaths due to flames, scalding and hot object contact under a single description. This is due to the space constraints of the data collection form. This limits the description of different fatal burn mechanisms, although it is expected that the major cause of burn deaths is due to open flames.

#### Denominator data

At the time of the study, the Census 2001 was the most comprehensive database available for Cape Town [15,16]. The population denominators were calculated for 2002, 2003 and 2004 based on a population growth model that takes into account the projected impact of HIV, as developed by the Actuarial Society of South Africa (ASSA) [17]. The ASSA model utilises fertility, mortality and migration information from the South African censuses, various surveys and vital statistics to provide estimates for population growth. The Cape Town's population growth for 2002, 2003 and 2004 was estimated for age, sex and population groups [17].

### Data analysis

**Figure 1:**
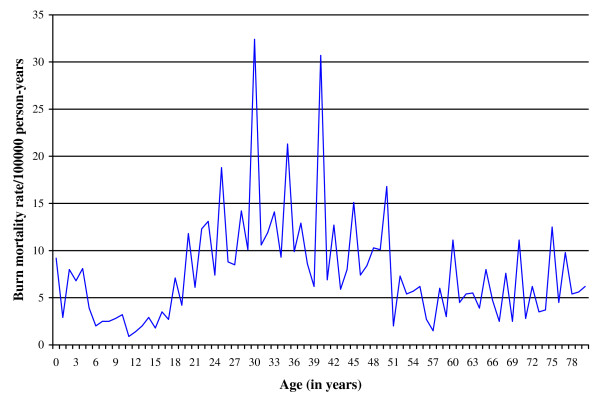
**Distribution of burn mortality by age, Cape Town, 2001 to 2004 (N = 1024)**.

Mortality rates were computed for all burns irrespective of type. Cases were stratified by age, sex and population group. In 62 cases, one or more of these variables were unknown. Age categories were developed based on the distribution of burns (see Figure 1) and approximate those generated by human development theorists [e.g. [18,19]]: 0 to 15; 16 to 24; 25 to 38; 39 to 50; and 51 years and older; and by childhood age groups (0 to 2; 3 to 6; 7 to 12; and 13 to 15 years). Three population groups were considered: black; coloured (referring to mixed heritage); and white/Asian. These terms were created through apartheid laws, but are still used, as they have social significance as a consequence of the profound impact of the earlier legislation. In the current analysis the white and Asian population groups were combined because of the more limited burn mortality reported for these groups, the relative demographic similarities between these groups, and the small number of Asians in Cape Town. Rates were calculated by relating the number of burn cases reported in the NIMSS over 2001 and 2004 [7] to the number of person-years of observation, i.e. the total number of years that each member of the study population was under observation, expressed per 100 000 per annum.

Mortality frequencies were reported by day, month and time of occurrence, and the proportion of fatalities above the legal blood alcohol limit was calculated by age category.

Stata (Version 7) [20] was used for the analysis to establish the significance of mortality rates and rate ratios (RR). A Poisson model was fitted to the data with age group, gender and population group as the main effects. Year as a main effect was also assessed, but was not statistically significant and therefore not included in the final model. Thus, all p-values presented in the results are corrected for the main effects. For the analysis of the 0-15 year olds, the following were the reference groups: 0-2 year olds, girls and blacks. For the all age categories analysis, the following were the reference groups: 25-38 year olds, women and blacks. P < 0.05 was used to indicate statistical significance.

The study was approved by the ethical review board of the South African Medical Research Council in November 2006.

## Results

### Injury distribution by age, sex and population group

The NIMSS data for Cape Town from 2001 to 2004 comprises 22 136 records for non-natural fatalities, 1024 of which were due to burn injuries [7]. A burn mortality rate of 7.9 per 100 000 person-years was reported in Cape Town over the study period. Figure 1 shows the distribution of the age of burn fatalities in Cape Town over the four-year period.

These cases included 988 burns (96.2%), 30 electrocutions (2.9%), and 9 deaths due to explosive blasts (0.9%). Most cases were accidental (94.5%), with few homicides (2.5%), suicides (1.1%), and of undetermined intent (1.8%). Two thirds of fatalities across the age spectrum (66.8%) occurred in the home, a pattern echoed for children (72.1%).

The distribution of burn mortality by age group, sex and population group is described in Table 1, reported according to the groups: children and all ages.

**Table 1:** Age specific distribution of burn mortality rates and incidence by sex and population group for 2001 to 2004 (N = 962)

	Burn mortality rates (95% CI) and Incidence (n)*					
				**Population group**		
				
**Age group (years)**	**Overall Burn Rate ** **and Incidence**	**Male Rate ** **and Incidence**	**Female Rate ** **and Incidence**	**Black**	**Coloured**	**White & Asian**

**Children**						

**0-2**	5.9 (4.3;8.0) (42)	6.7 (4.3;10.0) (24)	5.1 (3.0;8.1) (18)	9.0(6.0;13.1) (27)	4.6(2.6;7.7) (15)	n/a (0)**#**

**3-6**	4.7 (3.4;6.3) (41)	4.8 (2.8;7.0) (21)	4.6 (2.8;7.1) (20)	6.8(4.3;10.3) (23)	4.2(2.5;6.6) (18)	n/a (0)#

**7-12**	2.2 (1.5;3.2) (28)	3.0 (2.8;4.7) (19)	1.5 (0.7;2.8) (9)	4.5(2.7;7.1) (18)	1.5(0.7;2.8) (10)	n/a (0)#

**13-15**	2.2 (1.2;3.7) (14)	3.4 (1.5;5.7) (11)	0.9 (0.2;2.7) (3)	4.1(1.8;8.1) (8)	1.8(0.6;3.8) (6)	n/a (0)#

**Total**	3.6 (3.0;4.3) (125)	4.3(3.4;5.4) (75)	2.9(2.2;3.8) (50)	6.2(4.9;7.7) (76)	2.8(2.1;3.7) (49)	n/a (0)#

**All Ages**						

**0-15**	3.6(3.0;4.3) (125)	4.3(3.4;5.4) (75)	2.9(2.2;3.8) (50)	6.2(4.9;7.7) (76)	2.8(2.1;3.7) (49)	n/a (0)#

**16-24**	7.4(6.3;8.6) (158)	9.6(9.4;13.5) (102)	5.2(3.9;6.7) (56)	13.3(10.9;16.0) (107)	5.0(3.7;6.5) (50)	0.3(0.0;1.7) (1)

**25-38**	12.8(11.5;14.0) (396)	19.0(16.9;24.6) (292)	6.6(7.4;10.4) (104)	22.7(20.1;25.5) (279)	8.3(6.8;10.0) (112)	0.9(0.3;2.2) (5)

**39-50**	10.9(9.2;12.2) (194)	15.5(13.0;18.4) (135)	6.3(4.7;7.9) (59)	22.1(18.2;26.8) (107)	9.2(7.3;11.4) (82)	1.1(0.4;2.6) (5)

**51+**	5.2(4.1;6.3) (89)	6.4(4.8;8.5) (49)	4.1(2.9;5.6) (40)	13.0(9.1;17.9) (37)	5.4(3.9;7.3) (41)	1.6(0.8;2.9) (11)

**Total**	7.9(7.3;8.3) (962)	10.9(10.1;11.8) (653)	4.9(4.3;5.4) (309)	15.0(13.9;16.3) (606)	5.8(5.2;6.4) (334)	0.9(0.6;1.3) (22)

* Burn mortality rates described as mortality per 100 000 person-years, with 95% confidence intervals; **n **describes all cases reported over the 4 year period.

^# ^No fatal burn cases were recorded for white and Asian children in Cape Town over the 2001 to 2004 period.

Age specific reports were available for 962 cases. Among children, the highest rates occurred in the two youngest age categories, 0 to 2 and 3 to 6 years. The 0 to 2 year mortality rate was significantly higher than that for 7 to 12 (p < 0.001) and 13 to 15 year olds (p = 0.005). The 3 to 6 year olds had similar rates to that of the younger group (p = 0.346). The higher burn mortality rates amongst the younger groups occur across population groups, with especially high rates among very young black children. Overall, male childhood rates were nearly 50% higher than those for females (p = 0.041). The rates for black children were 50% higher than those for coloureds (p < 0.001). Rates for white/Asian children were not calculated because of the absence of cases for this period.

The distribution of mortality rates forms an inverted U-shape across all age categories. The overall childhood burn mortality rate of 3.6 per 100 000 person-years is significantly lower than those for all other age categories (p < 0.001). Fatal burn rates increase after childhood to peak in the 25 to 38 and 39 to 50 age groups, with significantly higher rates occurring in these two groups than at other ages (p < 0.001). Overall, fatal burn rates for blacks were significantly higher than that for coloureds, in turn higher than that for whites and Asians. Mortality rates for blacks were 17.8 times higher than that of whites and Asians (p < 0.001), with rates for coloureds 7.1 times higher than that of whites and Asians (p < 0.001). When comparing only blacks and coloureds, the rates for blacks were significantly higher than that of coloureds for all age groups (p < 0.001).

Table 2 reports on the male to female rate ratios by population and age group during childhood and over the life span. In childhood, more male than female cases were reported, except for the 3 to 6 year age group; with increasing differences reported in the two older groups of children. Table 2 furthermore reports on male to female rate ratios over the lifespan. In general, males sustained fatal rates 2.2 times more than that for females (p < 0.001). The male to female mortality rate ratios, irrespective of population group, are significantly higher for the16 to 24 year olds (1.9; p < 0.001), the 25 to 38 (2.8; p < 001) and the 39 to 50 year olds (2.5; p < 0.001). For all age groups, black and coloured males are most likely to sustain fatal burns. The overall significant male to female rate ratio pattern is repeated for blacks, while for coloureds there is significantly greater male mortality only in the 0 to 15 (2.2; p < 0.001), 25 to 38 (2.1; p < 0.001) and 39 to 50 year age groups (2.7; p < 0.001). Rates for whites/Asians were not calculated for the 0 to 38 age groups because of the small number of cases, but included for the analysis of the 39 to 51+ age groups.

**Table 2:** Male to female burn mortality rate ratios (with 95% CI) by population and age group (in years) during childhood and over the life span

**Population Group**	**Children (n = 125)**				**All ages (n = 962)**					
	**0-2**	**3-6**	**7-12**	**13-15**	**0-15**	**16-24**	**25-38**	**39-50**	**51+**	**All ages**
**Black**	1.2 (0.5;2.9)	0.6 (0.2;1.6)	1.6 (0.6;4.7)	3.1 (0.5;31.2)	1.2 (0.7;1.9)	2.1 (1.4;3.3)	3.2 (2.4;4.3)	2.5 (1.6;3.8)	1.6 (0.8;3.3)	2.3 (2.0;2.8)
**Coloured**	1.5 (0.5;5.0)	2.0 (0.7;6.4)	3.9 (0.8;38.0)	4.9 (0.6;233.3)	2.2 (1.2;4.4)	1.4 (0.8;2.6)	2.1 (1.4;3.1)	2.7 (1.6;4.4)	1.5 (0.8;2.8)	2.0 (1.6;2.5)
**Other**	n/a	n/a	n/a	n/a	n/a	n/a	n/a	1.6 (0.2;19)	1.5 (0.4;6.2)	2.3 (0.9;6.8)
**Total**	1.3 (0.7;2.6)	1.0 (0.5;2.0)	2.1 (0.9;5.2)	3.7 (1.0;20.5)	1.5 (1.0;2.2)	1.9 (1.3;2.6)	2.8 (2.3;3.6)	2.5 (1.8;3.5)	1.6 (1.0;2.4)	2.2 (2.0;2.6)

### Mortality distribution by circumstances of occurrence

There were 26 burn homicides over the four years (all with a specified age). Of these, 18 victims were male, with as many among coloureds and blacks (9 each) and none among whites and Asians. Nearly half of the homicides affected the 25 to 38 year olds (11 cases), 7 cases the 16 to 24 year olds and 4 cases the 39 to 50 year olds. Single homicide cases were reported for children and for persons over 51 years.

Table 3 presents the sex specific proportions of burn mortality by blood alcohol content (BAC) and by age group. BAC was available for 440 cases (52.6%) of the fatal burns population aged 16+ years and is described according to the legally prescribed limit for driving, i.e. <0.05 g/100 ml [21]. BAC levels above the legally prescribed levels were reported for 64.6% of all males and 60.0% of all females tested. The highest proportions of elevated BAC levels occurred for both males and females in the 25 to 38 year (75% and 68.6% of males and females respectively) and 39 to 50 year age groups (72.7% and 78.3%%). There was BAC information for 14 of the homicides; 6 (42.9%) were in excess of the legally prescribed levels.

**Table 3:** Sex specific proportions (%) of burn mortality by blood alcohol content (BAC) and by age (N = 467)

		**Age group (in years)**						
	**BAC Level**	**0-15**	**16-24**	**25-38**	**39-50**	**51+**	**Undetermined**	**All ages**
**Male**	≥ 0.05 g/100 ml*	0.0 (0)	54.0 (34)	75.0 (126)	72.7 (48)	56.2 (9)	58.8 (20)	64.6 (237)
	< 0.05 g/100 ml	100.0 (20)	46.0 (29)	25.0 (42)	27.3 (18)	43.8 (7)	41.2 (14)	35.4 (130)
**Female**	≥ 0.05 g/100 ml	0.0 (0)	43.8 (7)	68.6 (24)	78.3 (18)	50.0 (6)	71.4 (5)	60.0 (60)
	< 0.05 g/100 ml	100.0 (7)	56.2 (9)	31.4 (11)	21.7 (5)	50.0 (6)	28.6 (2)	40.0 (40)

* The Table represents the % of male and female fatalities that reported a BAC above or below the legal blood alcohol limit for driving in South Africa.

**Figure 2:**
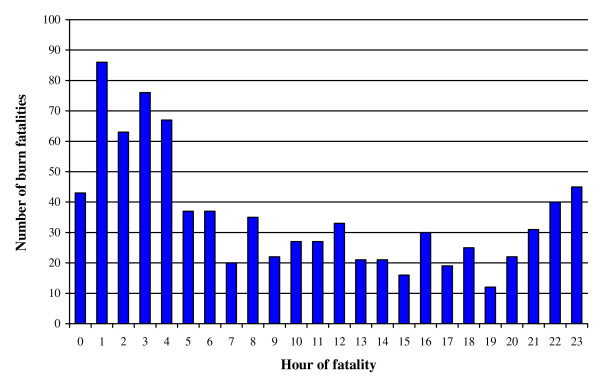
**Burn mortality by time of occurrence (N = 855)**.

Figure 2 describes the number of burn deaths by the time of death aggregated over the four year period. The study reports on time of death instead of time of injury. Death would probably have occurred at the time of injury for a majority of cases; however, some victims will have died hours or days after the injury itself, a bias to be noted when reading Figure 2. Most deaths were reported to have occurred in the early hours of the morning, with a third (33.9%; n = 290) occurring from 1 am to 4 am.

**Figure 3:**
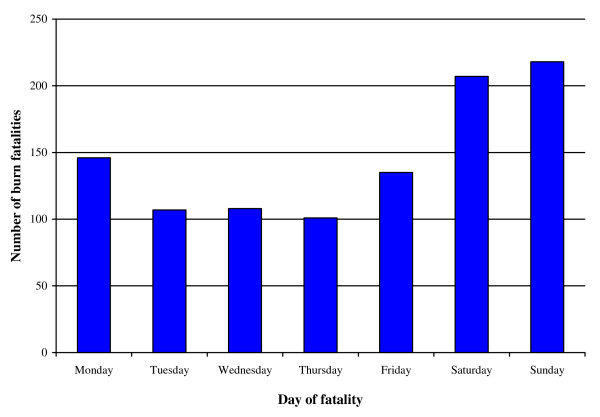
**Mortality by day of occurrence (N = 1022)**.

Figure 3 depicts mortality by the day of occurrence, aggregated for the four year period. Most deaths (41.4%; n = 423) occurring over the weekends. The number of deaths that occur on a day over the weekend are about double that for occurrences on days in the middle of the week.

**Figure 4:**
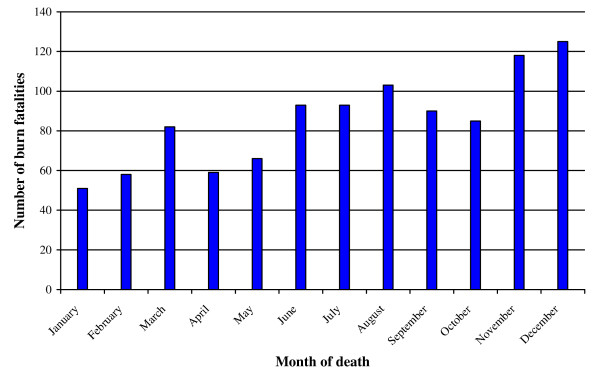
**Mortality by month of occurrence (N = 1023)**.

Figure 4 depicts the monthly fluctuation of burn mortality, again combined for the four year period. Cases steadily increase into the year, with the months of highest occurrence from June to December. A concentration of cases are reported in December (12.12%; n = 124) and November (11.44%; n = 117), coinciding with the end-of year vacation period, and August (9.97%; n = 102) and July (8.99%; n = 92) in the Cape Town winter (and the latter a vacation period for schools). A smaller concentration is reported in March (7.92%; n = 81), also a vacation period.

Table 4 presents the average seasonal rate of burn mortality per 100 000 person-years, with all fatal burns considered, irrespective of age. Fatal burn rates are highest in spring (September to November) (at 2.4 fatal burns per 100 000 person-years), significantly higher than the rates in autumn (1.7 per 100 000; p < 0.001) and in summer (1.9 per 100 000; p = 0.007), but similar to the rate in winter (2.3 per 100 000; p = 0.086).

**Table 4:** Distribution of burn mortality* by season (N = 1020)

**Season**	**Burn cases**	**Burn rate/100 000 (95% CI)**	**Rate ratio (95% CI)**	***P*-value (two-tailed)**
Summer (December to February)	231	1.9(1.6-2.1)	0.79 (0.66-0.94)	0.007
Autumn (March to May)	207	1.7(1.5-1.9)	0.71 (0.59-0.85)	<0.001
Winter (June to August)	289	2.3(2.0-2.6)	0.99 (0.84-1.16)	0.086
Spring (September to November)	293	2.4(2.2-2.7)	1.00	-

*Burn mortality rates described as mortality per 100 000 person-years.

## Discussion

### Main findings

In Cape Town burn mortality is concentrated amongst males aged between 25 and 50 years. These deaths are above all accidental, occurring most often in the home and in the early hours of the morning. They commonly take place over the weekend and other recreational periods across the year, with the expected concentration in the cold and wet months. Alcohol intoxication is a common denominator of the cases selected for testing. There is a smaller concentration of mortality amongst very young black children. This is consistent with burn morbidity studies in Cape Town [8,9] and mortality findings further afield [4].

The overall Cape Town burn mortality rate of 7.9 per 100 000 person years is higher than the world average of 5.0 per 100 000 and even the African Region one of 6.1 per 100 000 [1]. Despite its growing economy, the city remains beset by geographical pockets of poor housing conditions and impoverishment, closely associated in previous studies with child burn morbidity [9]. The adverse housing conditions are particularly related to the flammable materials used in the construction of the many informal homes or 'shacks' [6,22], the widespread use of portable kerosene stoves [23,24], and the storage of fossil fuels for heating and lighting, all individually associated with either greater burn hospitalization or mortality [24,25].

Impoverishment and poor socio economic status remain closely linked to population group in South Africa. It is thus unsurprising that burn mortality in Cape Town is concentrated amongst the black and to a lesser extent, the coloured population - as was the case for child burn morbidity [8,9]. Despite the economic progress of the country, these groups in general continue to report lower socio-economic status, including literacy rates, income levels, higher levels of household crowding and poorer overall health status [26], all factors associated with greater burn outcomes [27-29].

The highest rates of burn mortality in Cape Town are reported amongst adult males. This contrasts with findings from other studies and regions that have either highlighted a preponderance of burns during childhood (in LMICs and certain HICs) or amongst elderly populations (in HICs) [e.g. [3-5,30]], or reported a concentration of flame deaths amongst adult females, as in the South-East Asia/India region and parts of the Mediterranean [24,31]. The greater exposure for males observed for Cape Town may be due or exacerbated by the elevated levels of alcohol consumption reported [32]. In combination with smoking, a high level of alcohol consumption is associated with a greater occurrence of flame mortality [33] and linked for example to 73% of fire deaths in the United States [34]. The deaths in the current study occur in the early hours of the morning or during the late evening hours, when intoxicated drinkers would be the least easy to mobilise in terms of rapidly spreading house fires. Other reports indicate that cigarette-ignited fires often result in a period of smoldering before taking flame which then rapidly spreads, with resultant deaths typically reported between midnight and 06h00 [34]. The use in the home of kerosene, for kerosene or open flame heaters, would however result in especially rapid and devastating conflagrations [22,23].

Intoxication levels in this study are highest amongst males and in the age groups most affected by burn mortality. Men are especially involved in culturally sanctioned and binge drinking, which in recent years has been exacerbated by the increased availability and accessibility to commercial alcoholic beverages, increasing affluence, and the introduction of high alcohol content industrial brews [21,35]. Alcohol is widely used in Cape Town and is heavily embedded in everyday life, with the level of alcohol intoxication high compared to other South African cities [36].

However, more needs to be known about the living conditions and arrangements of adult men in Cape Town. There has been a significant increase in migration to the city in the 1990s, with considerable numbers of single migrant workers entering South African cities [37], and with many living in single male households. Single males are prone to risky drinking, particularly over weekends and during vacations; and in this study, at mid-year and at the end of year [21,35,38]. The study findings support the greater prioritisation of prevention activities that involve the implementation of strategies to reduce harmful drinking practices, provide alternatives to flammable housing materials, control kerosene heat sources, and promote the overall economic circumstances of impoverished households [6,22,23].

### Strengths and limitations

This study is based on one of the few systematic sources of burn mortality in Africa, an established, if urban-centred surveillance system [7,14,39]. The NIMSS has been positively rated for its simplicity, flexibility, sensitivity, positive predictive value and representativeness [13,14]. The system has limitations, including possibly selective BAC descriptions. The high rates of BAC reported in this study are however similar to those reported in other mortality studies [e.g. [5]] and South African hospital studies that utilised breath analyses [e.g. [32]]. The routine collection of alcohol data would enhance our quantification of the contribution of alcohol intoxication to burn mortality.

The study combines burn mortality with census data, and allows a socio-demographic mapping of the epidemiology of burn mortality across different strata of the population. As such, it contributes to the identification of targets for prevention and helps to generate hypotheses for further studies. The accuracy of the census data is however challenged by underestimations of the number of children below age five; the number of men relative to the number of women and the number in the white population; and over-estimates of the number of teenagers aged between 10 and 20. The first two are common features of censuses, particularly in LMICs [15,17]. The ASSA model adjusts for these features, thus controlling the over- or under-estimation of the reported burn mortality rates.

This study is also not able to clarify whether the excess mortality rates identified, as compared with others from the region, are a reflection of the adverse conditions in the city (due to a cohort effect) or an artefact of the better capture of cases in the NIMSS. This may need to be followed up more closely in a future study. Finally, there is no indication that morbidity studies would give the same type of age and population group distribution although we have reason to believe that the latter one would most likely be the same given the prior results on children [8,9]. It is also unclear how the group-specific male to female ratios would look.

## Conclusion

This study contributes to filling a knowledge gap by providing a socio-demographic mapping of burn mortality in an urban South African setting. In Cape Town, burn mortality is concentrated amongst the adult male population, with fewer cases than one might expect amongst children and to an extent the elderly. There are reasons to believe this over-representation is a reflection of detrimental living conditions (which adult males share with other segments of the population), life-style (where alcohol consumption is common) and poor socio-economic status (reflected in a population group bias). These contextual and individual determinants provide important targets for prevention interventions.

## Competing interests

The authors declare that they have no competing interests.

## Authors' contributions

LL and AVN devised the study. RL performed the statistical analysis. All authors made contributions to the interpretation of the data. AVN and LL drafted the manuscript. All authors have read and approved the final manuscript.

## Pre-publication history

The pre-publication history for this paper can be accessed here:



## References

[B1] Mock C, Peck M, Peden M, Krug E, Eds (2008). A WHO plan for burn prevention and care.

[B2] Burd A, Yuen C (2005). A global study of hospitalized paediatric burn patients. Burns.

[B3] World Health Organisation (2002). Injury; A leading cause of the global burden of disease.

[B4] Forjuoh SN (2006). Burns in low- and middle-income countries; A review of available literature on descriptive epidemiology, risk factors, treatment, and prevention. Burns.

[B5] Waller AE, Marshall SW, Langley JD (1998). Adult thermal injuries in New Zealand resulting in death and hospitalization. Burns.

[B6] Van Niekerk A (2006). Childhood burns in South Africa: Towards evidence for prevention action and policy. African Safety Promotion; A Journal of Injury and Violence Prevention.

[B7] Matzopoulos R (2005). A Profile of Fatal Injuries in South Africa: 6th Annual Report of the National Injury Mortality Surveillance System, 2004.

[B8] Van Niekerk A, Rode H, Laflamme L (2004). Incidence and patterns of childhood burn injuries in the Western Cape, South Africa. Burns.

[B9] Van Niekerk A, Reimers A, Laflamme L (2006). Area characteristics and determinants of childhood burn injury in Cape Town. Public Health.

[B10] Van Niekerk A, Seedat M, Menckel E, Laflamme L (2007). Caregiver experiences, contextualisations and understandings of the burn injury to their child. Accounts from low-income settings in South Africa. Child: Care, Health and Development.

[B11] Van Niekerk A (2007). Paediatric burn injuries in Cape Town, South Africa. PhD Thesis.

[B12] Bradshaw D, Timaeus IM, Jamison DT, Feachem RG, Makgoba MW, Bos ER, Baingana FK, Hofman KJ, Rogo KO (2006). Levels and trends of adult mortality. Disease and Mortality in Sub-Saharan Africa.

[B13] Matzopoulos R, Van Niekerk A, Marais S, Donson H (2002). A profile of fatal injuries in South Africa. African Safety Promotion; A Journal of Injury and Violence Prevention.

[B14] Butchart A, Peden M, Matzopoulos R, Phillips R, Burrows S, Bhagwandin N, Saayman G, Cooper G (2001). The South African National Non-natural Mortality Surveillance System-Rationale, pilot results and evaluation. South African Medical Journal.

[B15] Statistics South Africa (2003). Census 2001: Community profiles database [computer programme] Version 1 Cape Town.

[B16] (2006). South African Cities Network: State of the Cities Report. http://www.sacities.net/2006/pdfs/cities_2006_chapter3.pdf.

[B17] Dorrington RE (2005). Projection of the Population of the City of Cape Town, 2001-2021.

[B18] Duncan N, Van Niekerk A, Swartz L, de la Rey C, Duncan N (2004). Adulthood and Aging. Psychology.

[B19] Duncan N, Van Niekerk A, Mufamadi J, Nicholas L (2003). Developmental psychology: A life-span perspective. Psychology: An Introduction.

[B20] STATA (2005). Stata Reference Manual.

[B21] Parry CD (2005). South Africa: Alcohol today. Addiction.

[B22] Godwin Y, Hudson DA, Bloch CE (1996). Shack fires: a consequence of urban migration. Burns.

[B23] Peck MD, Kruger GE, Merwe AE Van Der, Godakumbura W, Ahuja RB (2008). Burns and fires from non-electric domestic appliances in low and middle income countries. Part 1. The scope of the problem. Burns.

[B24] Singh D, Jash PK, Tyagi S (1997). Recent trends in burn mortality in northwest India and its preventive aspects. Journal of Indian Academy of Forensic Medicine.

[B25] Furjuoh S, Guyer B, Strobino DM, Keyl PM, Diener-West M, Smith GS (1995). Risk factors for childhood burns: A case-control study of Ghanaian children. Journal of Epidemiology and Community Health.

[B26] Day C, Gray A, Ijumba P, Ntuli A, Barron P (2003). Health and related indicators. South African Health Review 2002.

[B27] Daisy S, Mostaque AK, Bari S, Khan AR, Karim S, Quamruzzaman Q (2001). Socioeconomic and cultural influence in the causation of burns in the urban children of Bangladesh. Journal of Burn Care and Rehabilitation.

[B28] Edelman LS (2007). Social and economic factors associated with the risk of burn injury. Burns.

[B29] Istre GR, McCoy MA, Osborn L, Barnard JJ, Bolton A (2001). Deaths and injuries from house fires. New England Journal of Medicine.

[B30] Bessey PQ, Arons RR, DiMaggio CJ, Yurt RW (2006). The vulnerabilities of age: burns in children and older adults. Surgery.

[B31] Panjeshahin M, Lari AR, Talei A, Shamsnia J, Alaghehbandan R (2001). Epidemiology and mortality of burns in the South West of Iran. Burns.

[B32] Plüddemann A, Parry CDH, Donson H, Sukhai A (2004). Alcohol use and trauma in Cape Town, Durban and Port Elizabeth, South Africa: 1999-2001. Injury Control and Safety Promotion.

[B33] Anwar M, Majumder S, Austin O, Phipps A (2005). Smoking, substance abuse, psychiatric history, and burns: Trends in adult patients. Journal of Burn Care & Rehabilitation.

[B34] Baker SP, O'Neill B, Ginsburg MJ, Li G (1992). The Injury Fact Book.

[B35] Parry CD, Plüddemann A, Steyn K, Bradshaw D, Norman R, Laubscher R (2005). Alcohol use in South Africa: findings from the first demographic and health survey (1998). Journal of Studies on Alcohol.

[B36] Groenewald P, Bradshaw D, Daniels J, Matzopoulos R, Bourne D, Shaikh N, Blease D, Zinyaktira N, Naledi T (2007). Cause of death and premature mortality in Cape Town, 2001-2004.

[B37] Cox KR, Hemson D, Todes A (2004). Urbanization in South Africa and the changing character of migrant labour. South African Geographical Journal.

[B38] Poznyak V, Peden M (2007). Alcohol and injury in emergency departments: summary of the report from the WHO collaborative study on alcohol and injuries.

[B39] Bowman B, Seedat M, Duncan N, Kobusingye O, Jamison DT, Feachem RG, Makgoba MW, Bos ER, Baingana FK, Hofman KJ, Rogo KO (2006). Violence and Injuries. Disease and Mortality in Sub-Saharan Africa.

